# A non-randomised controlled trial of the R&R2MHP cognitive skills program in high risk male offenders with severe mental illness

**DOI:** 10.1186/1471-244X-13-267

**Published:** 2013-10-18

**Authors:** Vivienne C-Y Yip, Gisli H Gudjonsson, Derek Perkins, Amie Doidge, Gareth Hopkin, Susan Young

**Affiliations:** 1 Department of Forensic and Neurodevelopmental Sciences, PO23, King’s College London, Institute of Psychiatry, De Crespigny Park, London SE5 8AF, UK; 2 Broadmoor Hospital, Crowthorne, UK; 3 Department of Psychology, PO77, King's College London, Henry Wellcome Building, De Crespigny Park, London SE5 8AF, UK

**Keywords:** Mentally disordered offenders, Treatment outcome, Reasoning & Rehabilitation, Cognitive skills

## Abstract

**Background:**

The growing popularity of offending behavior programs has led to the interest of whether such programs are effective with mentally disordered offenders. This study aimed to evaluate the effectiveness of the Reasoning and Rehabilitation program adapted for offenders with severe mental illness (R&R2 MHP).

**Methods:**

A sample of 59 adult high risk males detained in a high secure hospital completed questionnaires at baseline and post treatment to assess violent attitudes, anger, coping processes and social problem-solving. An informant measure of social and psychological functioning, including disruptive behavior, was completed by staff at the same time. The data of 30 patients who participated in the group condition were compared using intention to treat analysis with 29 controls who received treatment as usual.

**Results:**

80% of group participants completed the program. In contrast to controls, significant medium-large treatment effects were found at outcome on self-reported measures of violent attitudes, social problem-solving and coping processes. Improvements were endorsed by informant ratings of disruptive behavior, social and psychological functioning.

**Conclusions:**

The R&R2MHP had a comparatively low dropout rate and was effective in a sample of high risk mentally disordered offenders requiring detention in high security. Future research should use a randomized controlled design.

**Trial registration:**

Current Controlled Trials ACTRN12613000216718.

## Background

Individuals with severe mental illness (SMI) are at an increased risk of committing violent crimes. This is a robust finding supported by a variety of studies using a range of different methodologies including: population cohort studies [1-4]; community prevalence studies [5,6] and retrospective studies [7,8]. This is also evidenced by the high occupation of forensic inpatient beds by those who have SMI [9]. To alleviate this pressure and as prison and hospital populations rise, there is an increased demand for evidence-based treatments designed to reduce antisocial behaviors in those with SMI.

Research indicates that predictors of recidivism are similar for offenders with and without mental disorder [10,11]. These shared predictors include: maladaptive beliefs and attitudes supporting a criminal lifestyle [12,13] and poor social problem-solving ability [14-17]. Indeed, pro-criminal thinking has emerged as the strongest predictor for offending behavior [18-20]; others include institutional violence [21], poor psychosocial functioning and treatment engagement [22]. The association with anger is less clear; some research has reported it is a greater predictor of aggressive behavior than pro-criminal attitudes whereas other research suggests it is not necessarily associated with aggressive behavior [23,24].

Detainment is the most conventional method of disposal for those who commit severe criminal offences, yet there is paucity of research reviewing how offenders cope with their detainment and how it influences re-offending after release from prison. Zamble and Porporino [25] propose that an individual’s ability to cope with life circumstances is a critical factor for future adaptation. In their investigation of coping behaviors in imprisoned offenders, they found that offenders exhibited poor coping behaviors which maintained criminal behavior and moreover led to recidivism.

### Treating high risk offenders

Mentally disordered offenders (MDOs) with complex clinical needs and severe aggression are commonly detained in high secure hospitals. They are considered ‘high risk’ as they pose a grave and immediate danger to the public (Department of Health, 2006). Compared with those from low and medium secure hospitals, these patients have a diverse and complex range of psychiatric and criminogenic needs, often presenting with high rates of comorbidity and difficulty engaging with their clinical care [26,27].

Martinson [28] sparked debate after claiming that nothing worked in offender rehabilitation. This led to closer investigations of the efficacy of offender rehabilitation programs and has subsequently contributed to an amassing wealth of ‘what works’ research [29], which indicates that treatments targeted towards rehabilitating offenders are effective. Blud et al. [30] suggested that reducing recidivism was possible if interventions were appropriately designed, focused towards aims and delivered in a systematic manner.

Since then, a growing interest has developed in the effectiveness of Offending Behavior Programs (OBPs), the most common being the 22-session Enhanced Thinking Skills and the 36-session Reasoning and Rehabilitation [R&R] programs [31,32]. Both these OBPs employ a cognitive-behavioral paradigm which has been shown to be effective for reducing recidivism rates in juveniles and adult offenders [33,34]. However, these OBPs have not been designed to meet the complex needs of MDOs. Whilst a treatment effect was reported for clinical outcomes in a randomised control trial of the original R&R program, there was a 50% attrition rate of those allocated to the treatment condition [35]. This is important from a risk perspective, as research suggests that recidivism rates are elevated amongst non-completers [36-38]. Antisocial personality disorder, recent violence and psychopathy have been found to predict dropout [35].

High rates of treatment dropout call into question whether OBPs with a general offending focus are relevant and responsive to the needs of MDOs. The Risk Need Responsivity model suggests that structured and targeted programs should be delivered with the aim to match content and pace of treatment with specific offender characteristics [39]. Consistent with this theoretical paradigm, R&R has been adapted to meet the needs of MDOs (R&R2MHP) [40]. Firstly, the program duration was reduced from 36 to 16 sessions but supplemented by individual sessions provided by a mentor. The mentor role was introduced to improve treatment completion [41] and support the patient to rehearse and consolidate newly acquired skills and transfer them into daily activities. Secondly by inclusion of a module that addresses the executive dysfunction (such as attention and memory problems, poor organizational and planning skills) commonly experienced by many patients with SMI and that may interfere with their ability to effectively engage in OBPs [42].

Research has indicated that R&R2MHP is a feasible and effective treatment for MDOs [43,44]. A controlled multi-site study of patients detained in medium and low secure services reported a completion rate of 78% and positive clinical changes on self-reported measures of rational problem-solving, anger and attitudes towards violence. Improvement was supported by informant ratings of social and psychological functioning.

An evaluation of a similar program (R&R2 for ADHD Youths and Adults) in MDOs with a primary diagnosis of personality disorder detained in a high secure service has also shown a positive treatment effect, together with a 76% program completion rate [45]. However feasibility and effectiveness of R&R2MHP has yet to be investigated in high risk MDOs with a primary diagnosis of SMI. Furthermore, the treatment effect for improving coping processes in high risk MDOs has yet to be established. This study therefore evaluated the R&R2MHP in such patients using a quasi-experimental design comparing group participants with waiting list controls. The following research questions were investigated:

1. *How successful is the program in terms of retention of participants in a high secure setting?*

Consistent with the findings of Rees-Jones et al. [43], it was hypothesised that the completion rate would be greater than the 50% reported by Cullen et al. [35] in their study using the original 36 session R&R program.

2. *Is the program effective in patients detained in a high secure setting?*

Compared to controls, it was hypothesised that group participants would show significant improvements in violent attitudes, anger, coping processes, social problem-solving, disruptive behavior and social functioning.

## Methods

### Design and participants

**Figure 1:**
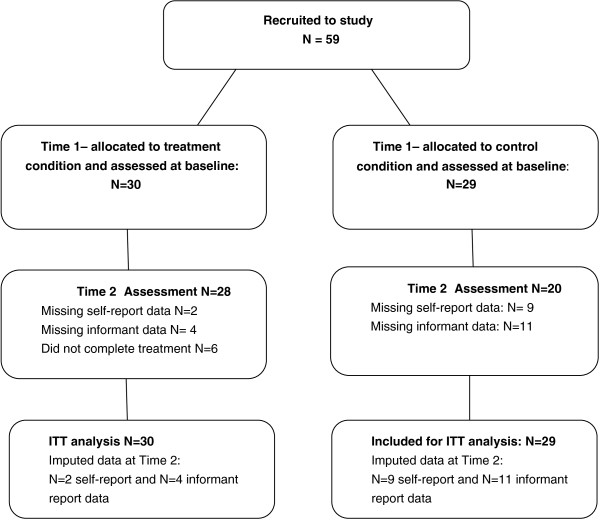
Flowchart of patient participation.

The quasi-experimental controlled study involved the participation of 59 male patients with SMI detained under the U.K. Mental Health Act (1983) in a high secure hospital setting. Thirty patients participated in the treatment condition (R&R2MHP). A total of five groups were delivered for this study. Their data were compared with that of 29 controls who received treatment as usual (TAU) (see Figure 1).

All participants were referred by their clinical team to attend the OBP. Inclusion criteria were age 18–65, a history of SMI (e.g. schizophrenia, schizoaffective disorder, bipolar disorder), a history of violent or antisocial behavior, no previous experience of participating in R&R, proficiency in English language. Exclusion criteria included intellectual disability, patients who were mentally unstable and/or who posed a risk of violence to the researcher.

### Intervention

R&R2MHP [40] is a manualised cognitive-behavioral intervention developed for antisocial youths and adults with mental health problems. It consists of 16, 90-minute, sessions which run on a weekly basis. It is a revised version of the original 36-session R&R program and aims to reduce anti-social behavior and attitudes and improve pro-social thinking, cognitive and problem-solving skills. The program employs a variety of methods to engage individuals such as individual group exercises, audiovisual material and workbooks which include homework assignments. The program consists of 5 treatment modules: (1) a neuro-cognitive model which introduces techniques to increase attentional control, impulse control, memory and constructive planning; (2) a problem-solving module which encourages problem identification, generation of multiple alternative solutions and consequential thinking; (3) an emotional control module which involves management of anxiety, anger and conflict; (4) a social skills module which aims to increase awareness of the thoughts and feelings of others; and (5) a critical reasoning module which aims to develop skills in the assessment and evaluation of information. The R&R2MHP program combines group and individual treatment. Individual treatment incorporates the use of a mentoring paradigm, whereby a mentor provides coaching sessions outside of group sessions. Their purpose is to facilitate the transfer of skills learned in group sessions into daily activities. Mentors are provided with written guidance on how to structure individual sessions. R&R2MHP facilitators were provided with training in how to deliver the program. Program integrity and consistency was ensured through (1) establishing a steering committee, where regular meetings were held and supervision was provided by SY (the program author); (2) random observations of group sessions by one of the program authors (SY); and (3) group supervision meetings of facilitators to prepare for sessions, process and discuss sessions that had been delivered and (4) regular meetings and supervision between program facilitators and mentors. Furthermore, R&R2MHP maximised program integrity by fostering consistency through its structured, manualised design.

### Treatment completion

A cut-off of ≥12 sessions was used to classify patients as completers, following the recommendation made by Cullen et al. [46] and representing 80% attendance of the programme.

### Treatment as usual

Control participants were not asked to refrain from attending interventions considered to be part of their usual treatment. However they were not permitted to attend the R&R2MHP program or similar cognitive skills interventions during the course of the study. Common psychosocial interventions included group and individual occupational, psycho-educational and cognitive-behavioral interventions for psychosis, personality disorder, violence, sex offending, substance misuse and relapse prevention.

### Measures

#### Baseline assessments

Demographic information, diagnosis, offence and admission history were obtained from clinical file reviews at the start of the study. In addition participants completed the Psychotic Symptoms Rating Scale (PSYRATS) [47] to assess severity of psychotic symptoms and the structured clinical interview for DSM-III-R personality disorders (SCID II) [48] to assess antisocial personality characteristics.

#### Outcome measures

The following measures were administered at baseline (Time 1) and repeated 16 weeks later post group (Time 2) to assess the primary (violent attitudes) and secondary outcomes (coping skills, social problem-solving, reaction to provocation, disruptive behavior and social functioning). The primary outcome measure was determined prior to commencement of the study. All measures were self-rated, except for the informant-rated Disruptive Behavior and Social Problem Scale (DBSP). The same informant (a member of the clinical team) completed the DBSP at Time 1 and Time 2.

1. Maudsley Violence Questionnaire (MVQ) [49] is a 56-item self-reported measure of cognitive style in relation to violent attitudes. It has two constructs: machismo (42 items which assess the extent to which the individual supports stereotypes of males as being dominant and tough) and acceptance (14 items which assess the acceptance of violence). A total score can be obtained by summing the scale scores (score range 0–56). High internal-consistency reliability for both constructs was estimated by Cronbach’s alpha (ranging between 0.74 and 0.91) in a male student sample [49] and it has specified differences between mentally disordered offenders [43,44].

2. Novaco Anger Scale - Provocation Inventory: Reaction to Provocation/Personal Affect Questionnaire (NAS-PI) [50] is a 48-item self-reported measure that assesses cognitive, arousal and behavioral domains of anger experience. Each item is rated on a 3-point Likert scale with higher scores indicating greater endorsement of anger (scores range between 16–48 for each domain); a total score can also be obtained by summing the domain scores (score range 48–144). The NAS-PI has good test-retest reliability (Cronbach’s alphas ranged from 0.78 to 0.91), concurrent validity, and the NAS total score has excellent internal-consistency (Cronbach’s alpha 0.92) [51,52]. It has been effective in predicting violence in MDOs [53] and discriminating between aggressive patients and non-clinical controls [54].

3. Ways of Coping Scale (WAYS) [55] is a 66-item self-reported measure of coping processes with responses. Participants are asked to recall a stressful situation that they have experienced in the past week. They are informed that this may be a situation that was difficult or troubling for them, either because they felt distressed about what happened, or because they had to use considerable effort to deal with the situation. They are told that the situation may have involved their family, work, friends or something else important to them. They are then asked to rate how often they used suggested behaviors to cope with that particular stressor on a 4-point Likert scale. The WAYS includes eight constructs, each with good internal consistency estimated by Conbach’s alpha as follows: (1) confrontive coping, 0.70; (2) distancing, 0.61; (3) self-controlling, 0.70; (4) social support, 0.76; (5) accepting responsibility, 0.66; (6) escape avoidance, 0.72; (7) planful problem-solving, 0.68; and (8) positive reappraisal, 0.68 [56]. In addition a total score can be obtained by summing the scale scores. The higher the score, the more effort the person applies to the coping process.

4. Social Problem-Solving Inventory-Revised: Short (SPSI-R: S) [14] is a 25-item self-reported questionnaire with responses rated on a 5-point Likert scale that assesses people’s ability to solve problems in everyday life. It consists of five subscales, two measuring problem-solving orientation (positive and negative) and three assessing problem-solving style (rational problem-solving, impulsivity/carelessness and avoidant). Scores range between 0–20 for each domain. An adjusted total score was obtained (score range 0–20) with higher scores reflecting better social problem-solving ability. This measure is reported to have high test-retest reliability (Cronbach’s alphas ranged from 0.68 to 0.91) and internal consistency (Cronbach’s alphas ranged from 0.69 to 0.95) [57].

5. Disruptive Behavior and Social Problems Scale (DBSP) [58] is an informant-rated questionnaire consisting of 14 statements rated on a 7-point Likert scale relating to patient’s behavior and social interactions. The scale consists of two factors, (1) disruptive behavior (score range 8–56), and (2) social and psychological functioning (score range 6–42). Higher scores indicate a greater degree of problems. Both factors have excellent or good internal consistency (Cronbach’s alpha 0.92 and 0.84 respectively).

### Procedure

Approval of the research project was given by Ealing and West London Research Ethics Committee. Patients meeting inclusion criteria and who were considered to be mentally stable and suitable for this intervention were referred by their clinical team. Referred patients were approached and invited to participate in the study. The treatment was not mandatory. A waiting-list controlled design was utilised and group allocation was determined by the order of referral. Once group capacity had been reached, the remaining patients were kept on a waiting-list for the following group. After providing informed consent, participants completed the self-reported measures at baseline (Time 1) and data were obtained from clinical file reviews. The DBSP was completed by a member of the clinical team who knew the patient well (most commonly the primary nurse). To reduce inter-rater differences, the same informant completed the questionnaire at both Time 1 and Time 2. Outcome measures were repeated again on completion of the group (Time 2). The timing between assessments was generally the same (16–18 weeks) for the R&R2MHP and TAU conditions. A total of 5 groups, each with 5–8 participants were delivered weekly. In addition group participants met with their mentor (a member of the clinical team, most commonly a health care or psychology assistant) between sessions. Session logs were completed to record group attendance. Researchers involved with data collection were not involved in the delivery of the treatment. Data were compiled centrally, input and scored using the Statistical Program for Social Sciences (SPSS) database.

### Statistical analysis

Descriptive statistics summarized demographics, clinical and forensic baseline characteristics. Independent-samples t tests were used to examine group differences at Time 1 (see Tables 1 and 2). Unadjusted mean scores and standard deviations on each of the outcome measures are provided in Table 3. A conservative intention to treat (ITT) analysis was used to assess outcome with missing data imputed by ‘last observation carried forward’. Figure 1 provides a flowchart of patient participation and shows that data were imputed at outcome for two treatment participants (7% of the treatment condition) and 9 control participants (31% of the control condition). This design (ITT) was adopted as this analysis is based on initial treatment intent rather than the treatment which was eventually administered [59]. Demographic and total score differences between the two groups at Time 1 were not significant for baseline and outcome measures, nevertheless in order to minimize error variance a two-tailed analysis of covariance (ANCOVA) was administered for each of the dependent variables measuring differences between the conditions in time using adjusted mean scores and standard deviations (see Table 3). The baseline Time 1 scores therefore served as covariates for the dependent Time 2 variables. The effect size was analyzed using Cohen’s *d* for efficacy measures.

**Table 1:** Participant characteristics comparing group participants (R&R2MHP) and controls (TAU) at baseline (Time 1)

	**R&R2MHP group**		**TAU group**		
	**N**	**M (SD)**	**N**	**M (SD)**	**t-value**
Age	30	37.93 (10.10)	29	38.72 (9.70)	−0.31
		Range 22-63		Range 20-66	
Number of previous admissions	30	4.07 (4.56)	29	3.24 (4.64)	0.69
		Range 0-15		Range 0-20	
Number of previous convictions	30	10.47 (14.45)	29	14.00 (20.30)	−0.77
		Range 0-65		Range 0-100	
PSYRATS total	30	7.87 (14.00)	29	2.79 (10.50)	1.57
SCID-II total	30	30.27 (12.48)	29	34.59 (9.78)	−1.48
MVQ total	30	15.83 (12.02)	29	18.14 (13.45)	−0.69
NAS-PI total	30	77.27 (16.78)	29	79.24 (15.74)	−0.47
WAYS total	30	46.90 (22.42)	29	42.82 (24.86)	0.66
SPSI total	30	12.39 (3.36)	29	11.57 (2.49)	1.07
DBSP total	26	35.59 (16.12)	27	39.00 (12.51)	−0.84

**Table 2:** Participant characteristics comparing group completers with non-completers at baseline (Time 1)

	**R&R2MHP completers**		**R&R2MHP Non-completers**		
	**N**	**M (SD)**	**N**	**M (SD)**	**t-value**
Age	24	36.67 (9.42)	6	43.00 (12.05)	−1.40
		Range = 22-59		Range = 31-63	
Number of previous admissions	24	4.08 (4.40)	6	4.00 (5.62)	0.04
		Range = 0-14		Range = 0-15	
Number of previous convictions	24	10.88 (15.16)	6	8.83 (12.25)	0.31
		Range = 0-65		Range = 0-33	
PSYRATS total	24	7.83 (14.58)	6	8.00 (12.65)	−0.03
SCID-II total	24	31.04 (12.08)	6	27.17 (14.76)	0.67
MVQ total	24	14.29 (11.20)	6	22.00 (14.30)	−1.43
NAS-PI total	24	77.04 (16.24)	6	78.17 (20.48)	−0.14
WAYS total	24	46.79 (20.96)	6	47.33 (29.94)	−0.05
SPSI total	24	12.65 (3.07)	6	11.37 (4.55)	0.83
DBSP total	21	32.67 (13.50)	5	48.40 (21.50)	−2.09*

*p < .05.

**Table 3:** Post-treatment ITT outcome data comparing R&R2MHP and TAU conditions

	**Baseline (Time 1)**		**Post-treatment (Time 2)**		**ITT Time 2 outcome**
	**R&R2MHP (N = 30)**	**TAU (N = 29)**	**R&R2MHP (N = 30)**	**TAU (N = 29)**	**F-value**
	**Mean (SD)**	**Mean (SD)**	**Mean (SD)**	**Mean (SD)**	**(Cohen’s *d*)**
MVQ Total	15.83 (12.02)	18.13 (13.45)	14.60 (11.22)	21.34 (14.67)	6.26 (.52)*
Machismo scale	8.60 (9.75)	10.79 (11.03)	8.00 (9.21)	12.86 (12.05)	4.33 (.45)*
Acceptance scale	7.23 (3.48)	7.34 (3.88)	6.60 (3.12)	8.48 (3.97)	7.62 (.53)**
NAS-PI total	77.27 (16.78)	79.24 (15.74)	80.00 (16.02)	81.00 (18.45)	0.01
Cognitive domain	27.90 (6.39)	28.07 (5.32)	28.33 (5.27)	28.59 (5.80)	0.02
Arousal domain	24.60 (6.03)	25.52 (6.12)	26.47 (7.07)	26.28 (6.63)	0.38
Behavior domain	24.77 (5.91)	25.66 (6.19)	25.20 (5.91)	26.14 (7.30)	0.06
WAYS total	46.90 (22.43)	42.83 (24.86)	61.50 (23.31)	33.41 (24.04)	35.56 (1.19)***
Confrontive coping	5.90 (3.22)	5.69 (3.26)	7.97 (3.77)	4.83 (3.26)	15.15 (0.89)***
Distancing coping	7.07 (3.59)	6.21 (3.60)	8.93 (4.22)	4.45 (3.58)	20.42 (1.14)***
Self-controlling coping	5.13 (2.53)	3.83 (2.56)	6.33 (2.89)	3.24 (2.84)	13.20 (1.08)***
Seeking social support coping	8.97 (7.92)	8.00 (5.70)	11.57 (7.10)	6.31 (4.80)	18.74 (0.87)***
Accepting responsibility coping	3.37 (2.34)	2.31 (2.59)	3.90 (2.50)	1.59 (2.38)	10.40 (0.95)**
Escape-avoidance coping	4.27 (3.23)	4.38 (3.91)	6.50 (3.03)	4.93 (3.57)	9.73 (0.47)**
Planful problem-solving coping	6.50 (3.62)	7.69 (6.99)	8.67 (3.35)	4.59 (4.20)	24.05 (1.07)***
Positive reappraisal coping	5.70 (4.11)	4.72 (4.38)	7.63 (4.63)	3.45 (4.35)	15.98 (0.93)***
SPSI-RS total	12.39 (3.36)	11.57 (2.49)	12.95 (3.32)	11.01 (3.84)	3.07
Positive problem orientation	11.20 (5.09)	9.41 (5.07)	12.13 (5.28)	7.86 (5.34)	10.73 (0.80)**
Negative problem orientation	6.43 (4.98)	6.41 (4.38)	6.00 (3.92)	5.59 (4.35)	0.22
Rational problem solving	10.50 (4.82)	8.14 (4.99)	10.80 (5.62)	6.17 (5.31)	6.72 (0.85)*
Impulsivity/carelessness	7.13 (4.88)	7.34 (5.13)	6.70 (4.34)	5.72 (5.21)	1.20
Avoidance style	6.17 (4.32)	5.90 (4.19)	5.57 (3.86)	4.00 (3.35)	4.20 (0.43)*
DBSP total	35.69 (16.12)	39.00 (12.51)	31.31 (12.96)	41.41 (13.34)	11.86 (0.77)**
Disruptive behavior	16.88 (10.43)	18.44 (10.04)	13.81 (8.11)	18.15 (9.76)	5.20 (0.48)*
Social and psychological	18.81 (7.77)	20.56 (6.89)	17.50 (7.93)	23.37 (6.93)	8.82 (0.79)**

*p < .05, **p < .01, ***p < .001.

In addition a post-hoc per protocol (PP) analysis was performed on the subgroup of participants for whom full data at Times 1 and 2 were available.

### Power calculation

Sample size was determined by a power calculation based on data obtained from a pilot study conducted by Young, Chick and Gudjonsson [44]. Calculations at 80% power with an alpha level of 0.05 suggested that 35 participants per group will be needed in order to detect a difference in the primary outcome measure with an effect size of 0.42 (pre-treatment mean 15.95 (SD = 10.83) and post-treatment mean 11.36 (SD = 10.53).

## Results

### Baseline characteristics

All participants had a history of severe mental illness, most commonly psychotic disorders, and violent offending including with index offences of homicide (37%), sexual violence (24%) and assault (39%). Independent t-tests showed that there were no significant differences between the R&R2MHP and TAU groups for age, previous number of admissions and convictions, current psychotic symptoms (PSYRATS), antisocial personality traits (SCID-II), and the total scores of outcome measures administered pre-treatment (see Table 1).

### Program completion rate

Of the 30 participants who started the R&R2MHP program, 24 completed the program giving a group completion rate of 80%. Group completers attended a mean of 14 sessions (SD 1.38; range 12–16) and the non-completers attended a mean of 7 sessions (SD 3.73; range 2–11).

Table 2 shows that there were no significant differences between completers and non-completers in age, number of previous convictions and admissions. Nor were significant differences found between R&R2MHP completers and non-completers on total scores of self-reported outcome measures administered at Time 1. However the non-completers were rated by informants to be significantly more behaviorally disturbed on the DBSP.

### Post-treatment outcome with ITT analysis

Table 3 presents unadjusted means and standard deviations for each of the outcome measures at baseline (Time 1) and outcome (Time 2) for both R&R2MHP and TAU. Results showed that being in the treatment group significantly reduced violent attitudes with medium effect for the Total MVQ score and Acceptance of Violence scale, and with small effect for the Machismo scale. No significant differences were found on NAS-PI scores assessing anger reactions to provocation.

All treatment effects for the WAYS coping processes were large. A significant improvement in effortful coping was found for the Total score, together with applying strategies relating to confrontive, distancing, applying self-control, seeking social support, accepting responsibility, escape-avoidance, planful problem-solving and positive reappraisal.

For social problem-solving, significant treatment effects were found for developing a more positive problem-solving orientation (large effect) and for engaging in a more rational problem-solving style (large effect) and avoidance problem-solving style (small effect). No significant differences were found between groups for the SPSI-RS Total score, negative problem solving orientation or impulsive/carelessness style.

Staff ratings of disruptive behavior and social/psychological functioning were significantly improved with a medium treatment effect for the DBSP Total score and the Social/Psychological scale, and a small effect for the Disruptive Behavior scale.

### Post-treatment outcome with PP analysis

A similar pattern of results was found when a PP analysis was conducted with the treatment effect increasing for all significant outcomes, now ranging between medium and large effect (see Table 4).

**Table 4:** Post-treatment PP outcome data comparing R&R2MHP and TAU conditions

	**Baseline (Time 1)**		**Post-treatment (Time 2)**		**PP Time 2 outcome**
	**R&R2MHP (N = 28)**	**TAU (N = 20)**	**R&R2MHP (N = 28)**	**TAU (N = 20)**	**F-value**
	**Mean (SD)**	**Mean (SD)**	**Mean (SD)**	**Mean (SD)**	**(Cohen’s *d*)**
MVQ Total	15.83 (12.02)	18.13 (13.45)	13.42 (10.52)	21.75 (15.56)	6.98 (0.63)*
Machismo scale	8.60 (9.75)	10.79 (11.03)	7.08 (8.66)	12.95 (13.26)	4.90 (0.52)*
Acceptance scale	7.23 (3.48)	7.34 (3.88)	6.35 (3.12)	8.80 (3.53)	8.75 (0.74)**
NAS-PI total	77.27 (16.78)	79.24 (15.74)	79.12 (15.63)	79.25 (19.75)	0.00
Cognitive domain	27.90 (6.39)	28.07 (5.32)	28.00 (5.41)	28.35 (6.47)	0.03
Arousal domain	24.60 (6.03)	25.52 (6.12)	26.31 (7.11)	25.45 (6.80)	0.26
Behavior domain	24.77 (5.91)	25.66 (6.19)	24.81 (5.44)	25.45 (7.59)	0.09
WAYS total	46.90 (22.43)	42.83 (24.86)	62.08 (22.66)	25.65 (18.64)	44.83 (1.76)***
Confrontive coping	5.90 (3.22)	5.69 (3.26)	8.08 (4.00)	4.00 (2.71)	16.13 (1.19)***
Distancing coping	7.07 (3.59)	6.21 (3.60)	9.00 (4.37)	3.40 (3.32)	23.32 (1.44)***
Self-controlling coping	5.13 (2.53)	3.83 (2.56)	6.65 (2.87)	2.55 (2.72)	16.56 (1.47)***
Seeking social support coping	8.97 (7.92)	8.00 (5.70)	10.81 (4.32)	4.85 (4.08)	26.54 (1.42)***
Accepting responsibility coping	3.37 (2.34)	2.31 (2.59)	4.12 (2.36)	.95 (1.88)	18.51 (1.49)***
Escape-avoidance coping	4.27 (3.23)	4.38 (3.91)	6.69 (3.07)	4.60 (2.37)	8.23 (0.76)**
Planful problem-solving coping	6.50 (3.62)	7.69 (6.99)	8.85 (3.51)	3.45 (3.38)	34.59 (1.57)***
Positive reappraisal coping	5.70 (4.11)	4.72 (4.38)	7.88 (4.68)	1.80 (3.22)	21.33 (1.51)***
SPSI-RS total	12.39 (3.36)	11.57 (2.49)	12.91 (3.26)	10.37 (4.05)	3.94
Positive problem orientation	11.20 (5.09)	9.41 (5.07)	11.96 (5.40)	6.65 (4.32)	13.24 (1.09)***
Negative problem orientation	6.43 (4.98)	6.41 (4.38)	6.12 (3.84)	5.70 (4.74)	0.26
Rational problem-solving	10.50 (4.82)	8.14 (4.99)	10.50 (5.95)	5.05 (4.89)	8.25 (1.00)**
Impulsivity/carelessness	7.13 (4.88)	7.34 (5.13)	6.42 (4.34)	5.65 (5.44)	1.08
Avoidance style	6.17 (4.32)	5.90 (4.19)	5.46 (3.47)	3.15 (2.68)	8.33 (0.75)**
DBSP total†	35.69 (16.12)	39.00 (12.51)	28.46 (9.11)	41.06 (13.99)	13.65 (1.07)***
Disruptive behavior†	16.88 (10.43)	18.44 (10.04)	11.46 (2.96)	17.06 (9.59)	8.14 (0.79)**
Social and psychological†	18.81 (7.77)	20.56 (6.89)	17.00 (8.77)	24.18 (7.90)	7.16 (0.86)*

*p < .05, **p < .01, ***p < .001.

† R&R2MHP N = 26 and TAU N = 1.

## Discussion

This study aimed to investigate the completion rate and effectiveness of the R&R2MHP program when delivered to high risk MDOs with SMI. We found a group completion rate of 80%; the 20% dropout rate is considerably lower than the 50% dropout rate reported by Cullen et al. [46] for delivery of the 36-session R&R program to MDOs. Hence the revisions that were made in R&R2 seem to have made the program more responsive to the needs of this population who are a complex group of offenders, presenting with severe mental illness and/or disorder, high rates of comorbidity, substance misuse, entrenched antisocial attitudes and rigid cognitive styles. The reduction in duration of the program together with the supplementation of a mentor, who provides individual sessions that aim to transfer skills from the group setting into daily activities, are likely to be important factors in limiting dropout [41].

These findings are consistent with results from a study evaluating the feasibility of R&R2MHP in low and medium secure settings, reporting a completion rate of 78% [43]. Cullen et al. caution that treatment dropout may be associated with a high risk status [35], suggesting that completion rates may be inflated by the inclusion of lower risk patients who are more advanced in the rehabilitation pathway. The low dropout rates observed in the current study, together with the 76% completion rate reported in a study of R&R2 delivered to high risk patients with a primary diagnosis of personality disorder [45], suggest that the revised program is feasible for treating patients at all levels of secure care.

It is essential to minimise dropout from OBPs as research has indicated that those who do not complete treatment display higher rates of re-offending compared to those who do not engage in treatment at all [36]. Conversely, those who complete treatment programs have lower reconviction rates compared to those who do not complete treatment [38]. In the current study, group non-completers were perceived by staff to be more disruptive and have more social problems than those who went on to complete the group. Their disruptive behaviour and social problems may make their ability to cope with group sessions more difficult and increase the likelihood of dropout.

The second aim of the current study was to evaluate the effectiveness of the program in high risk MDOs with SMI. As hypothesised, significant treatment effects were found at outcome for self-reported violent attitudes, coping processes and social problem-solving and for staff rated behavior on wards. A conservative ITT analysis found small to large treatment effects all of which improved (to medium and large) when a PP analysis was conducted.

As R&R2MHP’s primary aim is to reduce violent thinking and behavior, the present study used the self-report MVQ as a primary outcome measure to assess attitudes towards violence. Consistent with the findings of previous studies [43,45] a significant treatment effect was found for violent attitudes. This was endorsed by staff ratings of behavior on the wards indicating a significant reduction in social difficulties and disruptive behavior.

By contrast, Young, Hopkin et al. [45] reported significant results at outcome for NAS-PI anger cognitions in offenders with a primary diagnosis of personality disorder which were not supported by the current study or by Rees-Jones et al. [43], the latter studies including offenders with a history of SMI. Around half of the Young, Hopkin et al. [45] sample had borderline personality disorder suggesting that the measure may be more helpful in assessing emotionally labile patients.

Following treatment with the R&R2MHP program, an improvement in SPSI-RS problem-solving styles has previously been reported for rational thinking [43] and, using a similar program, for avoidance coping [45]. There was no significant treatment effect for impulsivity/carelessness style or the total problem-solving score, thus not supporting the improvement on these scales reported previously when a similar program was delivered to high risk personality disordered patients [45]. The current study was slightly underpowered which may explain lack of significance for these scales, or alternatively there may be some variability in outcomes for MDO patient populations, even for those detained at the same level of security. To our knowledge, this is the first time that a treatment effect has been reported indicating greater positive problem orientation in MDOs (large effect) and the finding indicates that participants in the treatment condition had developed more adaptive and constructive methods of social problem-solving compared with those in the control condition. The results provide further support for the existing empirical base which indicates that adaptive social problem-solving can be learnt, even by individuals who are considered to be high risk recidivists [34].

The largest treatment effect was found with respect to coping processes; all outcomes with the exception of one had a large effect size. In the pilot study, conducted on SMI offenders detained in high and medium secure hospital [44], the WAYS total score was not significant, although the pilot had a small control sample of only twelve patients. The present findings are however consistent with a previous study which assessed coping processes using a similar construct, the Coping Responses Inventory, and reported small to large effects in an evaluation of the longer 36-session R&R program [60]. The present findings indicate that the R&R2MHP program has a substantially significant effect on coping processes. The increase in confrontive coping is interesting and may not necessarily mean that patients take a more aggressive position, but are more prepared to assert themselves and have confidence to confront and deal with interpersonal problems. Similarly, the increase in distancing and avoidance coping may reflect that patients engage in techniques that help them to walk away from situations that are escalating and/or anger provoking.

The study has several limitations. Patients were referred as appropriate for the group treatment by their clinical team. Those who were not referred and the reasons for exclusion were not recorded. The largest exclusion categories were likely to be for reasons of mental instability and risk of violence to the researchers, thus reducing the generalisability of the study findings. It was slightly underpowered which may have resulted in lower effect sizes being identified. It has a quasi-experimental controlled design and group allocation was not randomized, thus in order to control for variance at baseline ANCOVA was used with baseline Time 1 scores covarying for the dependent outcome scores and a more conservative ITT analysis selected as the primary analysis.

A randomised methodology was not applied for pragmatic reasons because clinical teams did not wish to unnecessarily delay treatment. Whilst differences in baseline characteristics were not seen among those in the two arms of the trial, numbers were small and it is possible that other factors that influence outcome but were not measured differed between them. Given that those who received active treatment were the first to be referred, it is possible that they were more motivated to change their behavior. Missing data is common for a MDO sample, however we were unable to collect a substantial amount of informant Time 2 data due to high levels of staff turnover on wards. To avoid this in future, it is suggested that informant data is obtained with collective input from the clinical team, i.e. at ward round meetings. A record review of behavior on the ward (i.e. a record of critical incidents) was deemed unhelpful as previous review of such records has indicated a low baseline of incidents, with most patients having no incidents formally recorded. Future research should consider using a prospective assessment of aggression, such as the Staff Observation Aggression Scale Revised (SOAS-R0) [61], to obtain a record of every day, including minor, incidents.

The findings contribute to the growing evidence for treatment using R&R2MHP in different populations, however findings seem to differ between samples with differing patient characteristics and levels of security. Other characteristics that may influence outcome include IQ [62], self-esteem [63] impulsivity [64,65] and psychopathy [35]. It would be helpful if objective predictors of treatment outcome could be established, as this would mean that services could identify those who are likely to benefit from psychological interventions and those who require preparatory precursor treatments [66]. Outcome measures were administered by researchers who had not been involved in the delivery of the intervention, nevertheless a positive bias may still be present. Outcome measures consisted purely of clinical indicators. In particular, future research should include a cost-effective component in order that health economic outcomes can be assessed. There was no post-group follow up; other studies have reported an increase in treatment effects at 3-month follow-up, indicating that the program has a sustained influence over time [43,67]. Future research should use a randomized controlled design with follow-up data that includes objective assessments and reconviction data. It is of note that a meta-analysis of the original R&R programme, on which the revised R&R2 is based, found reductions in re-offending [34].

## Conclusions

The results of the present study support the use of group based cognitive-behavioral skills interventions with MDOs, and the R&R2MHP program has the advantage of being a manualised program which maximises program integrity. In spite of the aforesaid limitations, results from the present study contribute to its accruing evidence-base. Significant improvements were found in violent attitudes, coping processes and social problem-solving, and were endorsed by ratings of ward behavior. We are living in an era that demands cost-effective solutions for treating people with mental illness and group-work programs are a recognised target for development (The Sainsbury Centre for Mental Health, 2008). R&R2MHP is nearly half the duration of its predecessor and seems to be equally effective, hence representing a cost-effective program.

## Abbreviations

DBSP: Disruptive behavior and social problem scale; MDO: Mentally disordered offender; MVQ: Maudsley violence questionnaire; NAS-PI: Novaco anger scale and provocation inventory; OBP: Offending behavior programs; R&R: Reasoning and rehabilitation; R&R2 MHP: Reasoning and rehabilitation 2 for youths and adults with mental health problems; SMI: Severe mental illness; SPSI-RS: Social problem solving scale revised, short version; TAU: Treatment as usual; WAYS: Ways of coping scale.

## Competing interests

SY is a consultant for the Cognitive Centre of Canada and co-author of R&R2MHP. The remaining authors declare they have no competing interests.

## Authors’ contributions

SY, GG and DP contributed to the study design and management of the project, assisted by AD and GH. SY provided training in R&R2MHP. AD, GH and VY completed data collection and input. VY conducted the statistical analysis and completed a first draft of the manuscript. SY, GG and DP made revisions and edits to subsequent drafts. All authors have read and approved the manuscript.

## Pre-publication history

The pre-publication history for this paper can be accessed here:

http://www.biomedcentral.com/1471-244X/13/267/prepub
